# Brainhack Rome 2025: connecting the dots between structure and function

**DOI:** 10.1007/s00429-026-03211-8

**Published:** 2026-11-05

**Authors:** Matteo Mancini, Maria Guidi, Alessandra Maiuro, Marco Palombo, Edoardo d’Andrea, Luca Cairone, Federico Calesella, Yikang Cao, Manuela Carriero, Angelina Catrambone, Daniele Cirulli, Marida De Maria, Matteo De Matola, Emanuele Di Giorgio, Chiara Ercolano, Ottavia Falconi, Michela Fratini, Giulio Iannelli, Flavia Liporace, Lorenzo Massimi, Anna Notaro, Fabrizio Parente, Tiziana Pedale, Margherita Premi, Veronica Rossi, Darvin Shehu, Tiago da Silva Alves de Nogueira Simões, Taljinder Singh, Ginevra Sperandio, Leonardo Spitoni, Luca Taffarello, Pablo Villegas, Marco Zenari, Federico Giove

**Affiliations:** 1 Enrico Fermi Research Center, Rome, Italy; 2 Cardiff University Brain Research Imaging Centre, Cardiff University, Cardiff, UK; 3 Physics Department, Sapienza University of Rome, Rome, Italy; 4 Institute for Complex Systems, National Research Council, Rome, Italy; 5 School of Computer Science and Informatics, Cardiff University, Cardiff, UK; 6 Department of Anatomy, Histology, Forensic Medicine and Orthopaedics, Sapienza University of Rome, Rome, Italy; 7 Padova Neuroscience Center, University of Padova, Padova, Italy; 8 Institute for Advanced Biomedical Technologies, University “G. D’Annunzio”, Chieti, Italy; 9 Neuroscience Research Center, Magna Graecia University, Catanzaro, Italy; 10 DIIES, Mediterranea University, Reggio Calabria, Italy; 11 Center for Mind/Brain Sciences, University of Trento, Rovereto, TN Italy; 12 LUMSA University, Rome, Italy; 13 Neuroimaging Lab, Santa Lucia Foundation, Rome, Italy; 14 University of Rome “Roma Tre”, Rome, Italy; 15 CNR-Nanotec, Rome, Italy; 16 Department of Physics and Chemistry, University of Palermo, Palermo, Italy; 17 Medical Physics Department, Bambino Gesù Children’s Hospital, Rome, Italy; 18 Bocconi University, Milan, Italy; 19 Bocconi AI & Neuroscience Student Association (BAINSA), Milan, Italy; 20 Department of Physiology and Pharmacology, Sapienza University of Rome, Rome, Italy; 21 Department of Philosophy, Social Sciences & Education, University of Perugia, Perugia, Italy; 22 Neuroengineering and Medical Robotics Laboratory, Department of Electronics, Information and Bioengineering, Politecnico di Milano, Milan, Italy; 23 University “Niccolò Cusano”, Rome, Italy; 24 University of Campania “Luigi Vanvitelli”, Caserta, Italy; 25 Institute of Medical Psychology and Medical Sociology, Schleswig-Holstein, University Medical Center, Kiel University, Kiel, Germany; 26 Inserm U987, APHP, CHU Ambroise Paré, UVSQ, Paris-Saclay, Boulogne-Billancourt, France

**Keywords:** Brainhack, Hackathon, Neuroimaging, Complex systems, Brain, MRI

## Abstract

Brainhack events provide a bottom-up approach to tackle challenges in neuroscience. In 2025, the Enrico Fermi Research Center in Rome hosted its first Brainhack, with the goal of fostering interdisciplinary collaborations bringing together the physics and neuroscience perspectives on the study of brain structure and function. Structured in four days, it gave the chance to the participants to propose their own idea and shape a project based on it, leading to six research avenues: the combination of X-ray imaging and diffusion MRI algorithms to pioneer ultra-high-resolution tractography; multivariate approaches to study the shared factors between brain metabolism and microstructure estimated from MRI data; development of transfer entropy analysis-based tools for effective connectivity estimation in fMRI data; identification of brain states through Markov models; deep learning approaches to predict task EEG time courses; multi-scale network models to study functional and cerebrovascular data. Each project led to promising results and marked the beginning of new collaborations.

## Introduction

Brainhack is a collaborative meeting format that combines hackathon elements in a non-competitive formula to promote project-oriented collaborations and open science (Craddock et al. 2016). These events have become popular venues to foster inter-disciplinary collaborations within the realm of neuroscience research goals (Gau et al. 2021). In this direction, the Brainhack organised at the Enrico Fermi Research Center in Rome in April 2025 brought together 34 researchers with different backgrounds, ranging from physics to engineering and psychology, but with a common interest in understanding brain structure and function through data and models.

Modern brain research is increasingly shifting towards an interdisciplinary approach able to embrace different perspectives, ranging from the biological and clinical aspects to the complex system descriptions, which often share challenges, such as data processing and biophysical modelling. Despite this inherent interdisciplinary nature, effective cross-field collaborations are often hindered by the natural inclination to work more closely with people in local networks or in any case sharing the same background. This leads to field-specific language barriers, and ultimately to the so-called *research silos*. With this event, we wanted to take a step towards lowering these barriers in our own communities, in particular the ones working on neuroimaging and complex system perspectives. This goal was summed up by the title “connecting the dots between structure and function”, focusing on the idea of exploring this intersection between physics and neuroscience.

Brainhack events are particularly suited to this kind of goal: as they take inspiration from the hackathon style of work from software development environments, they can bring people together on the basis of research interest rather than background; at the same time, they do not have a competitive component and instead encourage sharing work across groups; the open structure, from project proposal to group formation and goal setting, ultimately serves the purpose of facilitating spontaneous collaborations. The event that has taken place in Rome in 2025 (https://brainhackrome.github.io/) was conceived on these premises.

To gather a diverse group of participants, the organization team used multiple channels to inform researchers about the event, contacting universities, research centres and conference mailing lists. Specifically, the team put together a list of universities and centres with research lines focused on the brain and proceeded to send inquiries through official addresses to circulate an informative email on the event – where possible, the organizers also leveraged direct contacts.

In the weeks before the actual event, anyone was able to start proposing project ideas through the event website and the associated Github repository – in this way, everyone could get feedback from the organisers and other participants, and some initial teams were formed. A dedicated channel was also arranged on the Mattermost platform, but most interactions took place through direct emails. On the first day, everyone who proposed a project made a short pitch, summing up the idea, a rough background and what they aimed to achieve during the hackathon, in order to build the working groups for the following days. The pitch session was followed by an informal Q&A session in an open space rather than in the conference hall where everyone was encouraged to reach out and to consider joining a team based on the interest or curiosity, without focusing too much on the overlap with their main activity. All the participants could then decide which project to join. In the four days of the event, the work focused on six projects that covered the topics of brain connectivity, microstructure, functional states and multimodal imaging. The general schedule for all the projects included spending most of the first day sharing ideas and expertise to make an action plan, dedicated the second and third days on focused work, and then summarizing their work in the form of presentation slides. The approach to projects included both individual work, dividing tasks between members, and pair programming style of work, to promote skill exchange and learn-by-doing. Despite the absence of dedicated educational sessions, to foster brainstorming and encourage a curious approach, four speakers were invited to give talks on project-related research activity, trying to balance general diversity as well as research fields. The topics – which included brain dynamics, network science, computational modelling, and quantitative MRI – were chosen trying to cover the vast landscape of approaches used in the brain structure and function research. On the last day, every group presented the work done and their results. In the following sections, we report a final summary for each of these projects.

## Adapting diffusion-weighted imaging algorithms to X-ray directional dark-field imaging

*Lorenzo Massimi*,* Marco Palombo*,* Alessandra Maiuro*,* Chiara Ercolano*,* Michela Fratini*.

### Introduction

The ability to determine fibre orientation is important in many fields, ranging from medical imaging to material science. In medical imaging, it is used to map the white matter pathways in the central nervous system, whose alterations are often associated with various diseases (Douaud et al. 2011; Schmahmann and Pandya 2009). In material science, fibre imaging helps to understand properties and failure mechanisms in carbon composites, widely used in aerospace and automotive applications (Heslehurst 2014). Fibre imaging is, however, challenging. Fibres can be directly visualized in 2D with microscopy or in 3D with X-ray microtomography, but the required resolution limits the field of view. This is a major drawback, especially in brain imaging, where long-range fibre bundles and their interactions are crucial for brain function (Martin-Herrero and Germain 2007). To overcome this limitation, diffusion-weighted imaging (DWI) infers fibre orientation from signal modulation allowing for larger field of view, specifically measuring the movement of water molecules – restricted when moving across fibres but less hindered along them. This anisotropy can be exploited to estimate both single and multiple fibre orientations within a voxel (Hasan et al. 2011; Tuch 2004). Similarly, X-ray small-angle and ultra-small-angle scattering (dark-field, DF) produce anisotropic signals, with maximum scattering perpendicular to the fibres (Endrizzi et al. 2014). This has also been exploited to retrieve fibre orientation in samples not compatible with MRI-based techniques, such as carbon composites. From a software point of view, MRI based directional techniques have advanced significantly, with standardized analysis pipelines and user-friendly software. In contrast, X-ray directional imaging still relies on custom-built code developed for specific samples or experiments, which makes it difficult to apply across different samples, instruments, or experimental conditions. Therefore, in this work, we test the feasibility of using the MRI software MRtrix3 (https://www.mrtrix.org - RRID: SCR_024123) to retrieve fibre information from X-ray data (Tournier et al. 2019).

### Methods

X-ray directional dark-field (DF) data were acquired at the I13 beamline of the Diamond Light Source using a conventional beam-tracking setup (Vittoria et al. 2015). The system employed an X-ray absorption mask with straight 20 μm apertures and a 80 μm pitch. To sample all regions covered by the mask septa, multiple images were collected with sub-pixel sample shifts. Angular modulation of the DF signal was probed by rotating the specimen in the detector plane from 0°to 180°in 10°steps. The sample consisted of carbon fibre sheets overlapped at different orientations, with each sheet approximately 0.2 mm thick. DF signals were retrieved from detector images using conventional beam-tracking phase retrieval, and subsequently averaged over a sliding 10 × 10 pixel window prior to MRtrix3 processing. Data were adapted for MRtrix3 by simulating an MRI volume with a single slice, consistent with the radiographic DF acquisition. A fake diffusion-weighting b-value file was provided, and the images were arranged into a 4D volume whose first two dimensions are the image, the third is a singleton dimension which would contain the third dimension when dealing with tomographic data, and the fourth contains the different angles. The response function was estimated from regions containing single fibres, with Lmax = 8 set as the maximum spherical harmonic order for deconvolution. The resulting orientation distribution functions (ODFs) were thresholded to extract fibre directions as fixels, which were then used as input for tractography analysis.

### Results and discussion

**Fig. 1:**
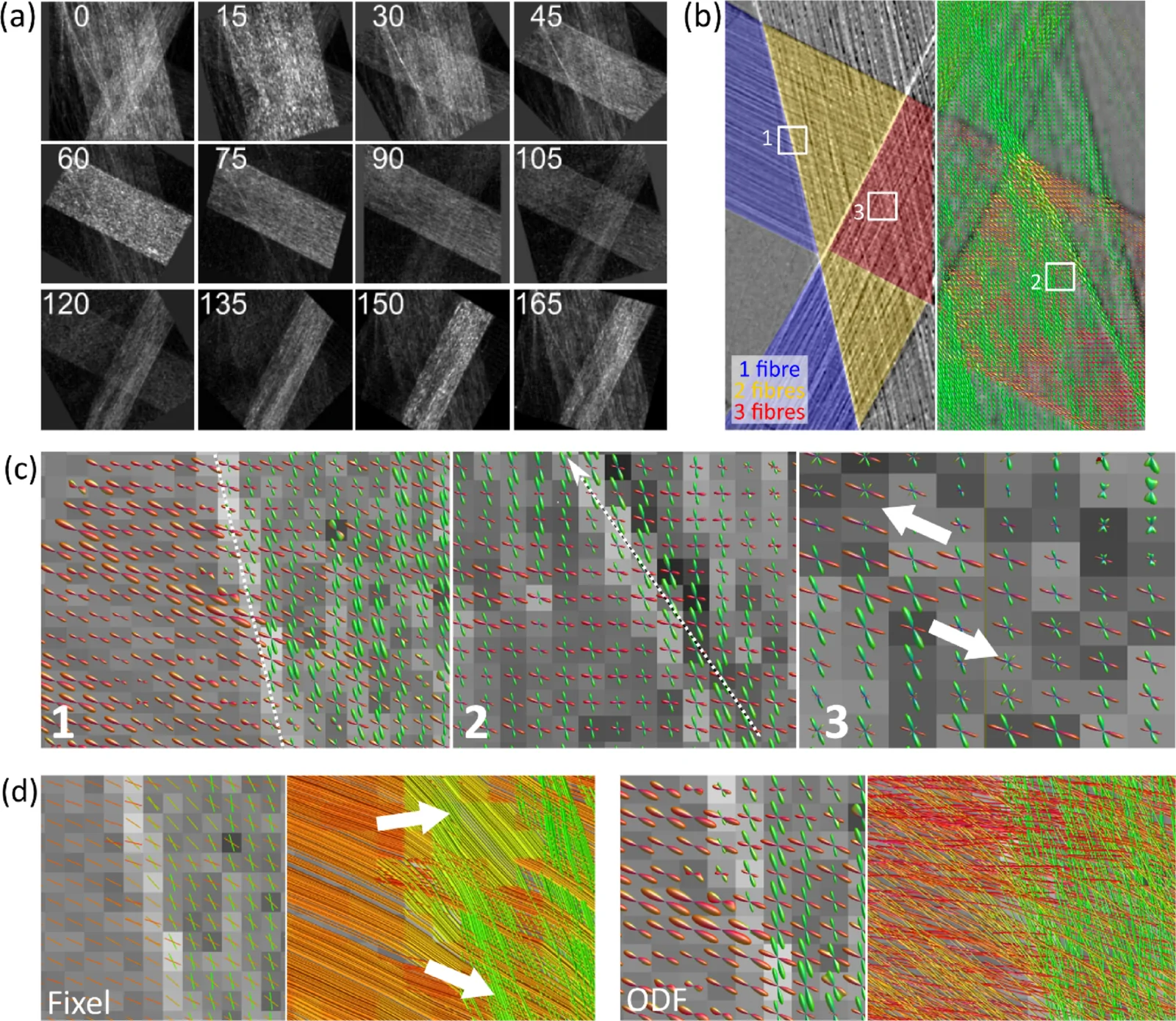
**a** X-ray DF images showing intensity modulation as a function of angular orientation with respect to the mask. **b** Refraction image with overlapped ODFs. The number of fibres is indicated with coloured overlays. **c** Magnified panels showing the ODFs in three selected regions of interest. **d** Results of tractography algorithms using deterministic and probabilistic fibre tracking

The main results are summarized in Fig. 1. The maximum signal in X-ray DF images occurs when fibres align with the mask aperture directions. In line with this observation, refraction images match overall the orientation distribution functions (ODFs) retrieved with spherical deconvolution, even in presence of overlapping fibres in different regions. When looking into more detail in ODF performances, crossings between one and two fibres produced two distinct ODF lobes in good agreement with directions measured from the refraction image (in Fig. 1c, about 103° and about 153° for region 1). In region 2, strong DF scattering from a fibre strand generates well-aligned lobes, while overlapping fibres produce ODFs of similar amplitude. With 3-fiber crossings, the correct number of fibres was still retrieved, though with lower accuracy. In terms of tractography, deterministic algorithms (FACT – fiber assignment by continuous tracking (Mori and Van Zijl 2002) reproduce straight tracts in single-fibre regions and crossings in two-fibre regions, but generate sharp orientation changes due to limited angular flexibility (see white arrows). Probabilistic tractography provides smoother and more realistic reconstructions by weighting connections according to ODF lobe amplitudes. Overall, ODFs from X-ray DF data match fibre orientations with excellent accuracy for single fibres and good accuracy for two-fibre crossings, while performance decreases for three fibres, reflecting known limitations of spherical deconvolution with more complex and noisy data. While these results were obtained on a phantom specimen, they demonstrate that MRI-based algorithms, such as those in MRtrix3, can be successfully adapted to X-ray scattering data, enabling reproducible analyses with extensively developed and standardized software instead of custom-built scripts. In addition, compared to existing geometrical models (Massimi et al. 2026), the use of spherical deconvolution has the advantage of not requiring an a priori assumption about the number of fibre layers within each pixel, representing a significant step forward compared to existing models.

## Connecting the dots between brain energy consumption and microstructure through latent space

*Manuela Carriero*,* Tiago da Silva Alves de Nogueira Simões*,* Edoardo d’Andrea*,* Flavia Liporace*,* Darvin Shehu*,* Taljinder Singh*.

### Background and goal

Brain functions are defined by specific energy consumption values, which are linked to their metabolic demands (Saberi et al. 2024), and by the cell types, that are reflected in the tissue microstructure (Ruqayya et al. 2022), which in turn is intrinsically linked to brain structure. Thus, studying the relationship between brain energy consumption and microstructure can bridge the gap between brain function and structure, allowing for healthy brain characterization. Currently, brain energy consumption and grey matter microstructural parameters are available thanks to calibrated fMRI (Chiarelli et al. 2022; Driver et al. 2024; Germuska and Wise 2019) and diffusion weighted imaging methods (Palombo et al. 2020).

How these data are related can be investigated using multivariate regression modelling. Partial least squares (PLS) regression (Hervé 2010) is capable of estimating model coefficients despite collinearity between variables, by finding orthogonal components, i.e. linear combination of variables, that maximize the covariance between the independent variables X and the dependent variables Y. PLS is similar to principal component analysis (PCA) (Gewers et al. 2021), whose components are obtained, instead, through variance maximization, without distinction of dependent and independent variables. Another output of PLS and PCA algorithms is the latent space, a representation of data in the component space. Being the means that allows the model to capture the underlying data patterns, latent space can reveal underlying patterns that, in the original space, are difficult to find. Although PCA and PLS latent spaces have been used for pattern detection for decades (Wold et al. 2001), their interpretation remains under investigation (Léger and Chatelain 2025; Muñoz et al. 2026).

The brain energy–microstructure relationship has been explored in prior studies, but primarily using computational modelling (Stone 2018) and invasive experiments in animals (Herculano-Houzel 2011). Non-invasive MRI methods, including apparent diffusion coefficient (ADC)-fMRI, have examined dynamic microstructural changes during functional tasks (De Riedmatten et al. 2025). However, a connection between resting-state energy consumption and tissue microstructure has yet to be investigated.

Here, we examine PCA and PLS latent-space properties and the relationship between brain energy consumption and microstructure, using calibrated fMRI and DWI-derived parameters.

### Methods

The MRI data were acquired at the Institute for Advanced Biomedical Technologies in Chieti, Italy (Caporale and Di Censo 2025). Brain energy consumption was represented by the cerebral metabolic rate of oxygen consumption (CMRO₂), which was estimated using the calibrated fMRI method and the biophysical models described by Chiarelli and colleagues (Chiarelli et al. 2022). The microstructural parameters considered were the soma and neurite density imaging (SANDI) model parameters (Palombo et al. 2020), which are the soma radius $\:{R}_{soma}$, the soma, neurite and extracellular signal fractions, respectively $\:{f}_{soma}$, $\:{f}_{neurite}$ and $\:{f}_{extra}$, and the extracellular and intracellular diffusivity, respectively $\:{D}_{e}$ and $\:{D}_{in}$. In this study, samples are brain regional medians of parameters, defined by the automated anatomical labelling atlas (Rolls et al. 2020). We assume CMRO_2_ to be the dependent variable Y and the six microstructural parameters as the independent variables X.

Under this assumption, data were represented in PCA and PLS latent spaces, respectively considering the first and second components and the first X and Y components. The loading scores of the first principal components, representing each variable contribution, were also plotted. Variables were z-scored.

Patterns in latent space were assessed using unsupervised density-based spatial clustering of applications with noise (DBScan) (Ester et al. 1996). MATLAB and Python scripts based on PCA, PLS and DBScan built-in functions are publicly available, together with a synthetic “toy” dataset to facilitate a conceptual understanding of the PCA and PLS latent spaces interpretation (Carriero and da Silva Alves de Nogueira Simões 2026).

### Results and discussion

**Fig. 2:**
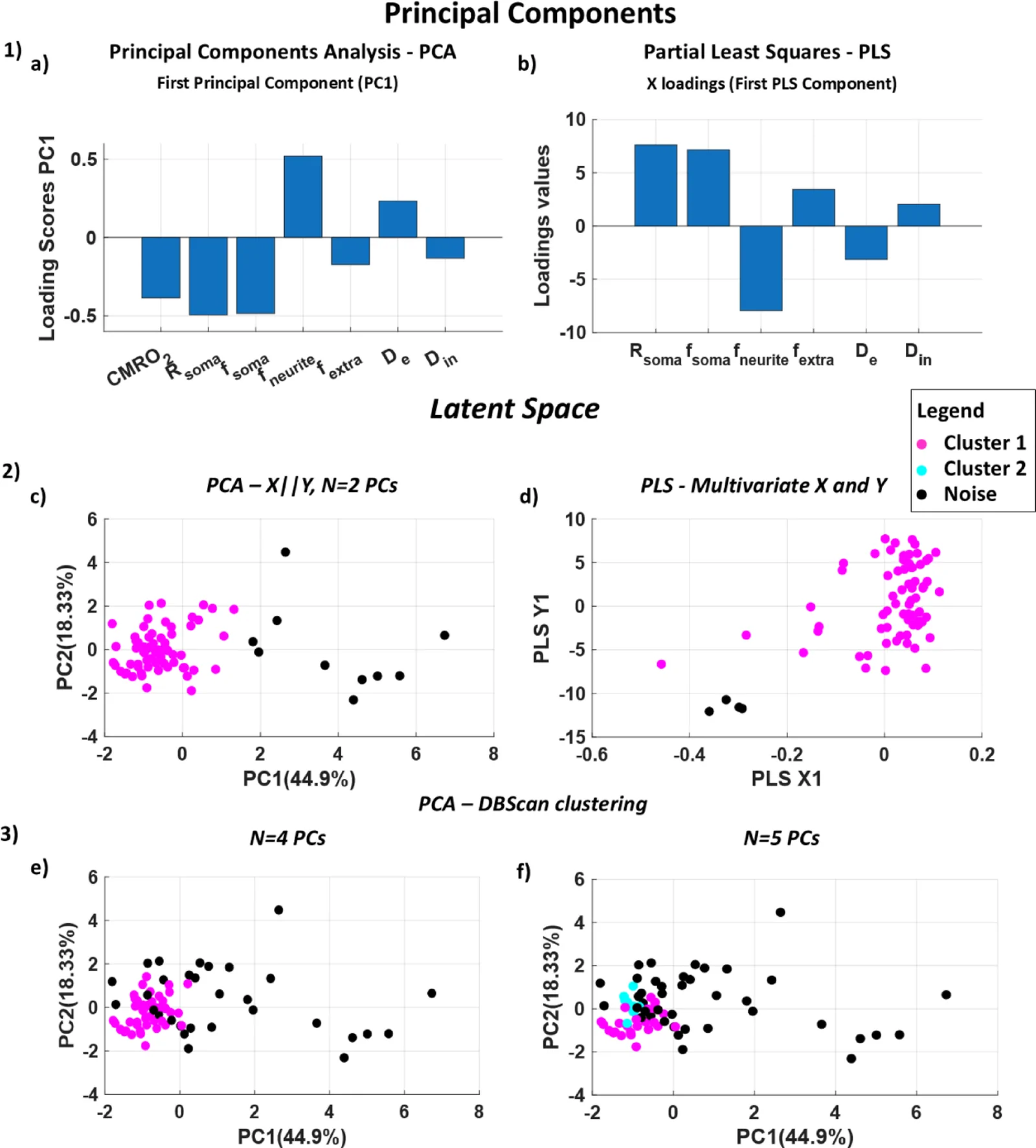
Analysis of principal components and brain regional parametric medians in the PCA and PLS latent spaces. Panel 1) Barplot of the loading scores relative to the first principal component of PCA (**a**) and PLS (**b**) method; Panel 2) Scatterplots of the MRI dataset in PCA (**c**) and PLS (**d**) latent spaces, clustered using the DBScan algorithm, with *N* = 2 components for the PCA case (**c**). Panel 3) DBScan results of the MRI dataset in PCA latent space considering *N* = 4 (**e**) and *N* = 5 (**f**) components. The DBScan maximum Euclidean distance between two samples belonging to the same cluster was set to $\:\epsilon\:=1$, and the minimum number of samples required to form a cluster * n=5*

Figure 2 shows the results of the analysis of PCA and PLS principal components (Panel 1) and the regional medians of MRI parameters in the PCA and PLS latent spaces (Panels 2–3).

We can notice that $\:CMR{O}_{2}$, $\:{R}_{soma}$, $\:{f}_{soma}$ and $\:{f}_{neurite}$ are the variables having the highest loading scores in magnitude, with opposite sign for $\:{f}_{neurite}$ with respect to $\:CMR{O}_{2}$, $\:{R}_{soma}$ and $\:{f}_{soma}$. The highest loading scores in magnitude are observed for the $\:{R}_{soma}$, $\:{f}_{soma}$ and $\:{f}_{neurite}$ parameters. Hence, similarly to the PCA analysis, $\:{R}_{soma}$, $\:{f}_{soma}$ and $\:{f}_{neurite}$ are the variables which contribute most to the first PLS latent component. This is in line with the fact that diffusivity parameters ($\:{D}_{in}$ and $\:{D}_{e}$) are known to be contaminated by noise (Schiavi et al. 2023), suggesting that PCA and PLS loading scores are, respectively, capturing only weak signal variance and spurious covariance between the $\:CMR{O}_{2}$ and the diffusivity metrics.

The regional medians of MRI parameters in the PCA and PLS latent spaces (Fig. 2 panel 2) show clear separation along the diagonal in PLS latent space, differently from PCA. Thus, patterns in the latent space depend on the function to be maximized as already observed in works comparing PCA and PLS methods (Godoy et al. 2014). Interestingly, we observed that the samples in the black cluster were concentrated within a narrow range for $\:CMR{O}_{2}$, $\:{R}_{soma}$, $\:{f}_{soma}$ and $\:{f}_{neurite}$, whereas they were more dispersed for the other parameters. This observation is consistent with the high contributions of the $\:CMR{O}_{2}$, $\:{R}_{soma}$, $\:{f}_{soma}$ and $\:{f}_{neurite}$ variables to the first PCA and PLS components. Finally, increasing the number of components (Fig. 2 panel 3) leads to the identification of a higher number of clusters.

Thus, we demonstrated that meaningful patterns exist within the MRI dataset by exploiting the structure of the latent space. We note, however, that in PCA, directions of high variance may reflect either true biological variability or increased noise variance (Sanguinetti et al. 2005), while in PLS, high covariance between predictor and response spaces may potentially arise from correlations between noise components in (X) and (Y) (Trygg and Wold 2003). Noise-aware approaches could improve the results (Lee et al. 1990; R. D. Phillips et al. 2009).

Nevertheless, the observation that the patterns are primarily structured according to CMRO₂, $\:{R}_{\mathrm{s}\mathrm{o}\mathrm{m}\mathrm{a}}$, $\:{f}_{\mathrm{s}\mathrm{o}\mathrm{m}\mathrm{a}}$, and $\:{f}_{\mathrm{n}\mathrm{e}\mathrm{u}\mathrm{r}\mathrm{i}\mathrm{t}\mathrm{e}}$is plausible, given that the soma and neurite compartments are expected to be closely associated with CMRO₂. Overall, these findings support the existence of a relationship between brain metabolism and microstructure, which in turn suggests a link between the functional and structural organization of the brain.

Future analyses will investigate the robustness of the MRI dataset patterns in latent space by assessing their dependence on variable selection and the tuning of hyperparameters within the unsupervised clustering algorithm. These analyses may ultimately allow the function–structure relationship to be described through a mathematical model capable of discriminating between clusters.

## Developing a MATLAB toolbox for transfer entropy analysis: a data-driven approach to measuring effective connectivity with fMRI data

*Fabrizio Parente*,* Tiziana Pedale*,* Luca Cairone*,* Yikang Cao*,* Veronica Rossi*.

### Background and goal

Revealing effective communications between brain regions from fMRI data remains a core challenge in neuroscience, addressed by methods like Dynamic Causal Modeling (DCM) (Friston 2011) and Granger causality (Seth et al. 2015). Here, we proposed a non-parametric approach based on Transfer Entropy (TE) (Schreiber 2000; Vicente et al. 2010), which estimates how the state of two regions at one time influences the future state of either region without statistical assumptions.

We developed a model to estimate resting-state fMRI effective connectivity by optimizing threshold definition, normalization, and null-model selection. Finally, we applied it to real fMRI data to estimate effective connectivity within the well-known medial prefrontal cortex (mPFC)–amygdala (AMY)–hippocampus (HP) circuit (Feng et al. 2016).

### Methods and results

Resting-state fMRI data were acquired from 42 healthy participants (25 ± 4 years; 23 females/19 males) with the following parameters: 3 × 3 mm in-plane resolution, 2.4 mm slice thickness, TR = 1.1 s, TE = 30 ms, and 322 volumes. The study was approved by the local Ethics Committee (Prot. CE/PROG.901), and written informed consent was obtained from all participants. Images were preprocessed with fMRIPrep (Esteban et al. 2019), including spatial normalization to MNI space and 8-mm FWHM Gaussian smoothing. BOLD time series were extracted with the CONN toolbox (Whitfield-Gabrieli and Nieto-Castanon 2012): from nine Harvard–Oxford ROIs: frontal medial cortex (defined as mPFC), bilateral AMY, HP, thalamus (TH), and intracalcarine cortex (ICC), with TH and ICC included as additional regions to extend the mPFC–AMY–HP circuit.

**Fig. 3:**
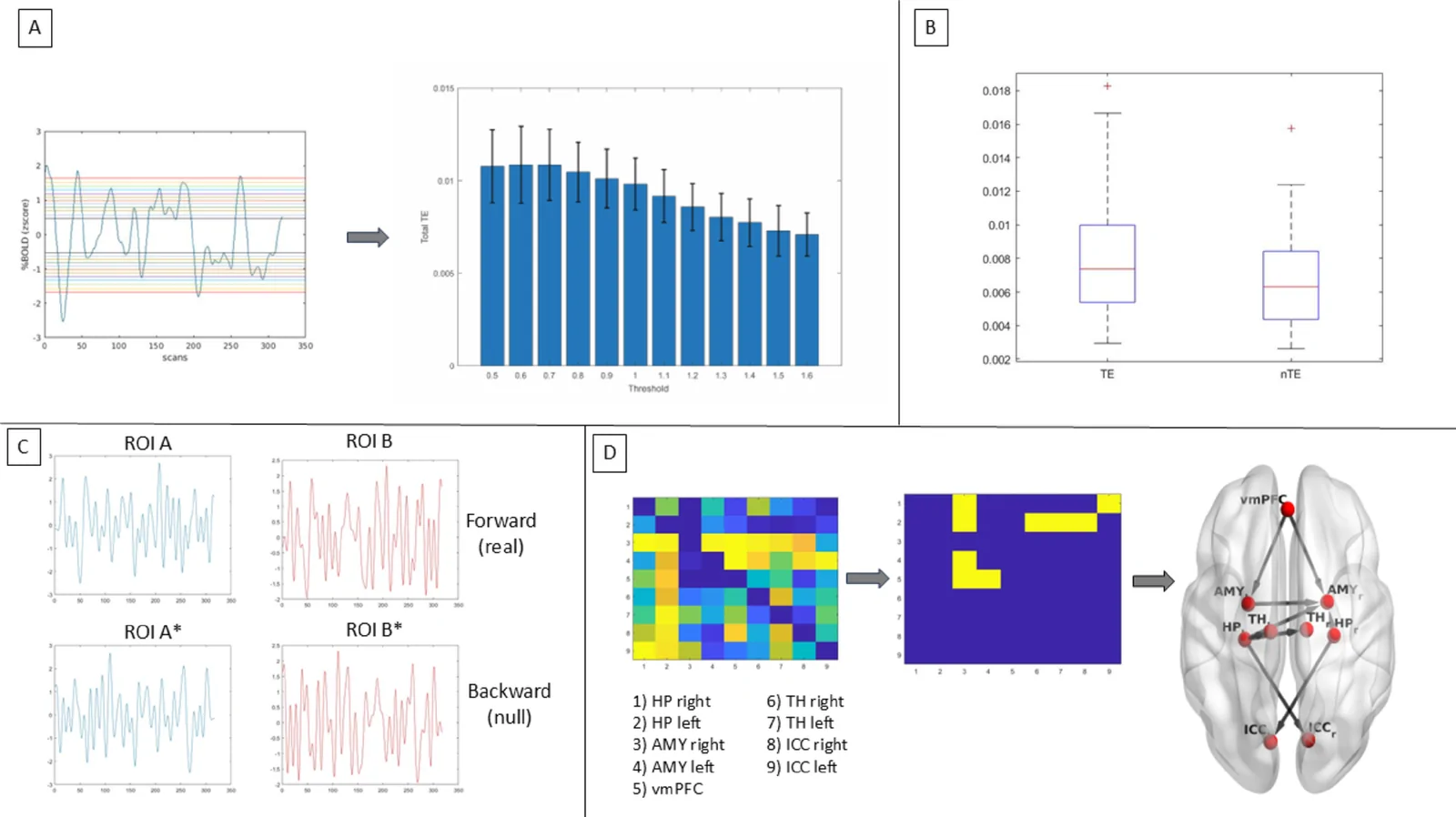
**A** Left side, the thresholding method to discretise the BOLD signal in single events; right side, bar plot of the estimated TE for each threshold; **B** Box plot comparing raw TE values with normalized TE (nTE) values, highlighting reduced variability after normalization; **C** Example of real (forward) vs. null (backward) signals used for the backward method; **D** Estimated ROI-to-ROI effective connectivity. Yellow boxes indicate statistically significant connections. Right: Brain representation of the identified circuit

We discretized continuous BOLD time series (Parente and Colosimo 2021) by applying 12 symmetric amplitude thresholds (± 0.5 to ± 1.6 SD, step 0.1), converting each signal into three values: 1 (above positive threshold), -1 (below negative), or 0 (in between) (Fig. 3a). To estimate causal relation, we used the ROI time series and calculated TE values by the following equation:

$$\:{T}_{A\:\to\:\:B}=\:\sum\:P({B}_{n+1};\:{A}_{n}^{\left(k\right)};\:{B}_{n}^{\left(l\right)})\mathrm{ln}\frac{P\left({B}_{n+1}|{A}_{n}^{\left(k\right)};{B}_{n}^{\left(l\right)}\right)}{P\left({B}_{n+1}|{B}_{n}^{\left(k\right)}\right)}$$

where the state of ROI A and B is specified by *n* = the current time step; *n* + 1 = the following time step; *k* and *l* = number of time steps before *n* for A and B, respectively (in our case both are set to 0).

#### Exploring thresholds

To explore the impact of different thresholds on TE values, ANOVA was performed on the total information estimated (i.e., summing all connections between ROIs) for each subject and the 12 thresholds. It shows a main effect of threshold (p-value < 0.00001) (Fig. 3^3^). The post-hoc analysis showed that TE values can be divided into three different clusters: highest amount of estimated information at [0.5–0.8]; intermediate amount at [0.9–1.2]; lower amount at [1.3–1.6]. The maximum peak, chosen for further analysis, was at 0.7 SD.

#### Normalization

Among many methods to overcome the normalization of TE values (Choi 2014), we used the one proposed by Gourévitch and Eggermontv (Gourévitch and Eggermontv 2007):

$$\:{nT}_{A\:\to\:\:B}=\:\frac{{T}_{A\:\to\:\:B}}{{H\left({B}_{t}\right|B}_{t-1\:})}$$

where H(B_t_|B_t−1_) is the entropy of the probability that ROI B is in a particular event (1,0, -1) at time t, given the probability that the same regions were characterized by another event at time t-1.

Figure 3b shows the amount of total variability with the normalized TE (nTE) vs. the raw ones. The reduced variability in nTE confirmed improved robustness of TE after this step.

#### Null model

Two methods were compared: (1) random shuffling of the fMRI time sequence (Lancaster et al. 2018), (2) backward time (Shi and Li 2024). The first method resulted in the loss of the natural dynamics of the BOLD signal, making the null time-series incomparable with the real one. While the second method maintains the features of the BOLD signal and only affects the temporal relationship between ROIs (Fig. 3c). We selected this latter and used two-sample t-tests to compare real (forward) vs. null (backward) signals for each of the 72 possible ROI-to-ROI connections.

#### Assessing the model

The application of these steps on real fMRI showed direct influences of mPFC on bilateral AMY, in agreement with previous findings (Belleau et al. 2018; Motzkin et al. 2015). Moreover, left HP appeared to influence right AMY and TH bilaterally. Right HP solely influenced right AMY. Finally, each HP side influenced contralateral ICC, and left AMY influenced the right one (Fig. 3d).

### Discussion

We developed an efficient pipeline for TE analysis by optimizing threshold selection for signal discretization, applying normalization to improve the robustness of TE estimates, and using the backward method for validation. Application to resting-state fMRI data yielded results consistent with previous studies (Belleau et al. 2018; Motzkin et al. 2015). These findings represent the first step towards an open-source MATLAB tool for estimating effective connectivity from fMRI data.

### Availability of supporting data

The code and test data are publicly available (Parente 2026).

## Hidden Markov models and task-based fMRI: dynamic brain patterns of emotional processing across psychiatric disorders

*Federico Calesella*,* Luca Taffarello*,* Marco Zenari*,* Ginevra Sperandio*,* Leonardo Spitoni*.

### Background

Disturbances in emotional processing and social cognition are hallmark features of many psychiatric disorders, consistently linked to aberrant activity and connectivity within the cortico-limbic network (Etkin and Wager 2007; Kaiser et al. 2015; M. L. Phillips et al. 2003). Traditional functional magnetic resonance imaging (fMRI) analyses typically emphasize mean activation or static connectivity, thus overlooking temporal dynamics. Hidden Markov Models (HMMs) provide a complementary framework for understanding brain activity by modeling it as a dynamical system that transitions between discrete recurrent states. These states, defined by unique spatial and temporal profiles, offer interpretable descriptions of transient brain dynamics and enable the detection of reproducible patterns of network activity (Baker et al. 2014; Taghia et al. 2017; Vidaurre et al. 2017). Beyond resting state, HMMs have been used to decode task-evoked dynamics and link transient states to behaviour (Taghia et al. 2017; Vidaurre et al. 2018), with altered occupancy, dwell time and transitions reported across psychiatric conditions.

### Goal

This study aimed to characterize dynamic brain states engaged during emotional processing using HMMs, and to assess how these states differ between psychiatric patients and healthy controls (HC).

### Methods

In order to do so, we employed the open dataset from the Transdiagnostic Connectome Project (*N* = 219), including 90 HC and 129 patients encompassing 15 distinct psychiatric conditions (Chopra et al. 2025). Participants underwent fMRI acquisitions while completing an emotional face-matching task (Hariri et al. 2002). In this task, participants are presented with a target face displaying an emotional expression (typically anger or fear) and are instructed to select, from two simultaneously presented probe faces, the one that matches the target’s expression. This emotion-matching condition is contrasted with a control condition in which participants match geometric shapes, thereby controlling for general perceptual and motor demands. The fMRI data were already preprocessed according to the HCP processing guidelines, and a high-pass temporal filter was applied. Regional BOLD signals were extracted with the 434-region whole-brain parcellation of the Transdiagnostic Connectome Project (Chopra et al. 2025), combining 400 cortical parcels from a homotopic update of the Schaefer atlas (Yan et al. 2023), subcortical parcels from the Melbourne Subcortex Atlas (Tian et al. 2020), and cerebellar regions. The timeseries of the 434 brain parcels were concatenated across subjects and spatially reduced via principal component analysis. The top 15 principal components were selected for the analyses, based on the elbow criterion. Since we retained a large number of components, we opted to fit a Gaussian HMM to reduce the number of parameters to be estimated. The number of states of the HMM was optimized based on the log-likelihood, over a set of possible values in the range between 2 and 14, yielding 5 states as the optimal value. For each time point, posterior probabilities of state occupancy were computed, enabling derivation of transition matrices and the frequency of state co-occurrence with experimental conditions, as well as each state’s dwell time and occupancy frequency. Group differences between HC and patients were assessed with Mann-Whitney U tests (Bonferroni-corrected, *p* < 0.05). The code is publicly available (Zenari 2026).

### Results

**Fig. 4:**
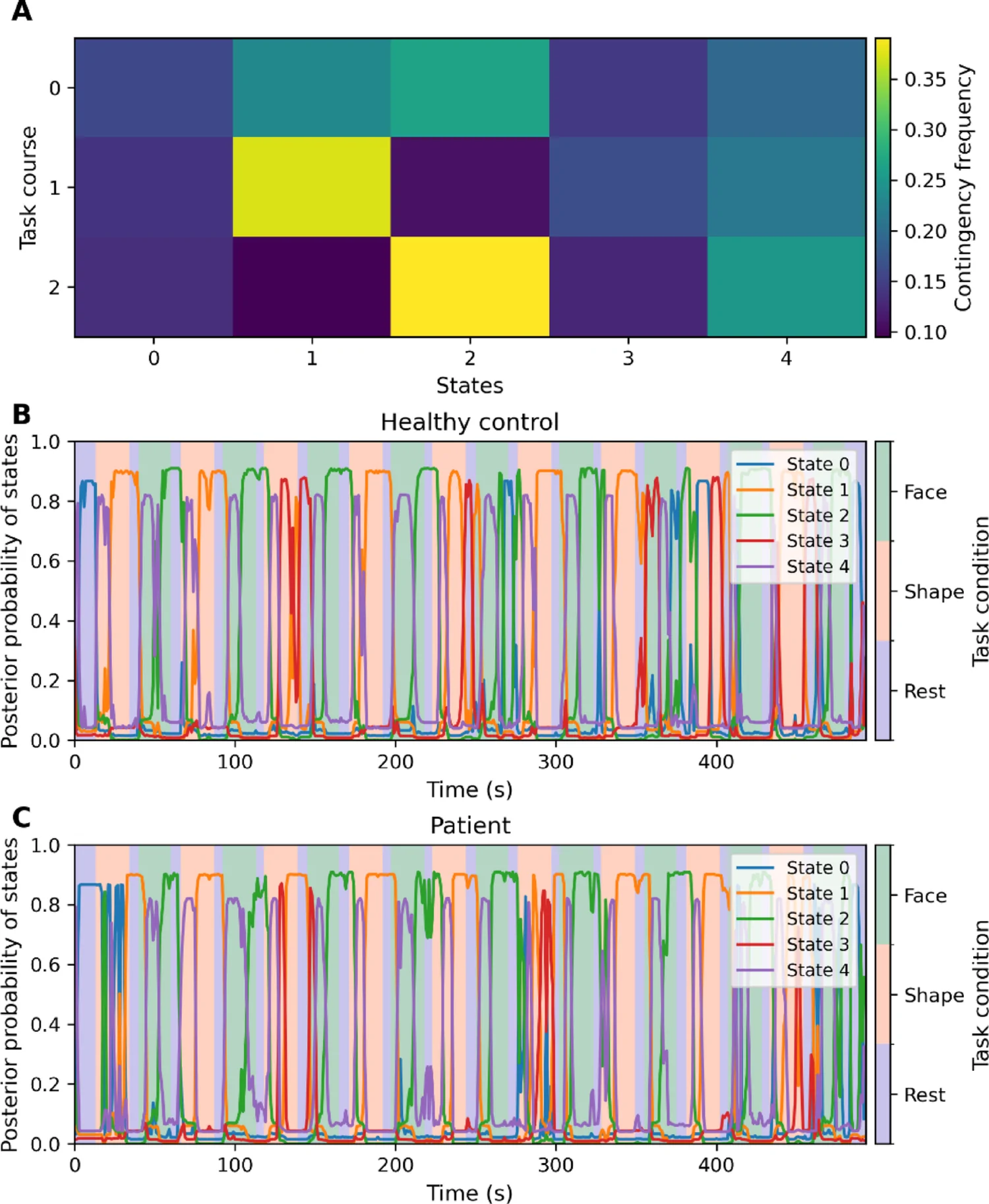
**A** Adjacency matrix indicating co-occurrences between states and experimental conditions. Experimental conditions coded as: 0 = no task; 1 = control (shapes) condition; 2 = experimental (faces) condition. **B** Posterior probabilities of states in an example healthy control subject. Background colours indicate the experimental condition. **C** Posterior probabilities of states in an example patient. Background colours indicate the experimental condition

The adjacency matrix between the states and the experimental conditions revealed relatively high co-occurrence of State-1 with the control condition and of State-2 with the emotional condition (Fig. 4). The other states did not show any specific overlap with the experimental conditions. The dwell time of State-2 was the only measure that showed a significant difference between HC and patients, with patients spending more time in this state (U = 1120701; *p* = 0.001). No significant differences were found for the other states and the occupancy frequency.

### Discussion

These results indicate that HMMs can correctly differentiate dynamic fMRI patterns underlying the processing of neutral compared to emotional stimuli. Notably, once triggered, the state related to emotional processing persisted longer in psychiatric patients, possibly reflecting a prolonged emotional processing, which could be caused by an abnormal emotional reactivity and an impaired inhibitory control over emotional activation. Overall, this work provided a fundamental step towards identifying dynamic, rather than static, brain patterns related to emotional processing using HMMs and characterizing these patterns in pathological conditions. However, further methodological refinements are required to improve the reliability and interpretability of the results, such as: (i) the implementation of a data-driven optimization of the number of components; (ii) performing model selection based on measures that account for the generalization ability, such as cross-validated log-likelihood or Bayesian information; (iii) accounting for the hemodynamic response function and other possible sources of noise (e.g., movement); (iv) the estimation of subject-specific measures, instead of group-level aggregation; (v) the analysis of the means and covariances of the states to provide a neurocognitive interpretation of the states.

## “I know what you will do”: forecasting motor behaviour from EEG time series

*Anna Notaro*,* Emanuele Di Giorgio*,* Matteo Mancini*,* Matteo De Matola*.

### Background

Electroencephalography (EEG) is a non-invasive technique for measuring the electrical activity of pyramidal cells in the most superficial layers of the cerebral cortex (Jackson and Bolger 2014). Thanks to its high temporal resolution, EEG is particularly useful for studying fast-varying phenomena. Traditionally, researchers have leveraged this feature to capture millisecond-scale responses to exogenous events, such as sensory stimuli, using EEG as a mere readout of brain activity. Recent literature has suggested a second possibility: forecasting the EEG signal to predict brain activity in the immediate future. This approach has found application in the field of brain stimulation, where forecasting EEG oscillations in given frequency bands has allowed researchers to schedule stimulation pulses in an intelligent fashion, using the phase of ongoing oscillations as a control signal (Zrenner et al. 2018). While groundbreaking, such research has been relying entirely on a single forecasting model, univariate linear autoregression (uLAR), which might be underpowered with respect to the multivariate and nonlinear nature of raw EEG signals (De Matola and Miniussi 2025). In fact, the univariate and linear nature of uLAR circumscribes its capabilities to the case of one EEG channel that generates an approximately linear signal, like the one generated by a given oscillation in a very short time interval. More powerful models might forecast more complex signals in longer time intervals, predicting the entire (i.e., non-band-filtered) EEG signal with an advance of hundreds of milliseconds. In this respect, deep learning (DL) algorithms might be a promising avenue, as suggested by outstanding results in other domains and by two recent studies that have successfully applied DL solutions to electrophysiological signals (Csaky et al. 2024; Pankka et al. 2025).

### Goal

This project aimed to use DL to forecast lightly preprocessed EEG data acquired during a simple motor task, with potential applications to brain state-dependent stimulation and motor BCIs.

### Methods

We used the WAY-EEG-GAL dataset (Luciw et al. 2014b), which includes 32-channel EEG recordings from 12 participants performing a simple grasp-and-lift task. Given our interest in motor signals, we retained only the 14 electrodes overlying the main sensorimotor areas (F3, Fz, F4, FC5, FC1, FC2, FC6, C3, Cz, C4, CP5, CP1, CP2, CP6) to reduce the dataset’s dimensionality. In order to remove slow drifts and high-frequency noise, data were filtered with a zero-phase Butterworth band-pass filter with cut-off frequencies of 0.5 Hz and 40 Hz. Subsequently, an Independent Component Analysis (ICA) with automatic component labelling (Pion-Tonachini et al. 2019) was run to isolate and discard eye and muscle signals. The output of this pipeline was a collection of nine data matrices for each of the 12 subjects, representing lightly preprocessed continuous signals from the 14 channels of interest. Those signals were discretised into 1500-ms windows centred on the expected start of the grasp-and-lift movement, corresponding to the moment when experimental subjects saw a “go” signal. Within those windows, the first half ([-750 ms, 0 ms]) was treated as the measured past (covariate), while the second half ([0 ms, 750 ms]) was treated as the future to forecast (regressor). Our forecasting model was a variation of WaveNet (van den Oord et al. 2016), a generative model that was originally developed for audio time series. WaveNet uses a stack of dilated causal convolutions to process samples from the distant past while keeping a limited parameter count and preserving strict temporal causality. During the Brainhack, the model was trained to minimise the mean squared error (MSE) between the true and forecasted futures. In a post-event follow-up, the same model was re-trained to minimise a variation of MSE that explicitly penalises large amplitude mismatches and temporal shifts between the true and forecasted signals.

### Results

**Fig. 5:**
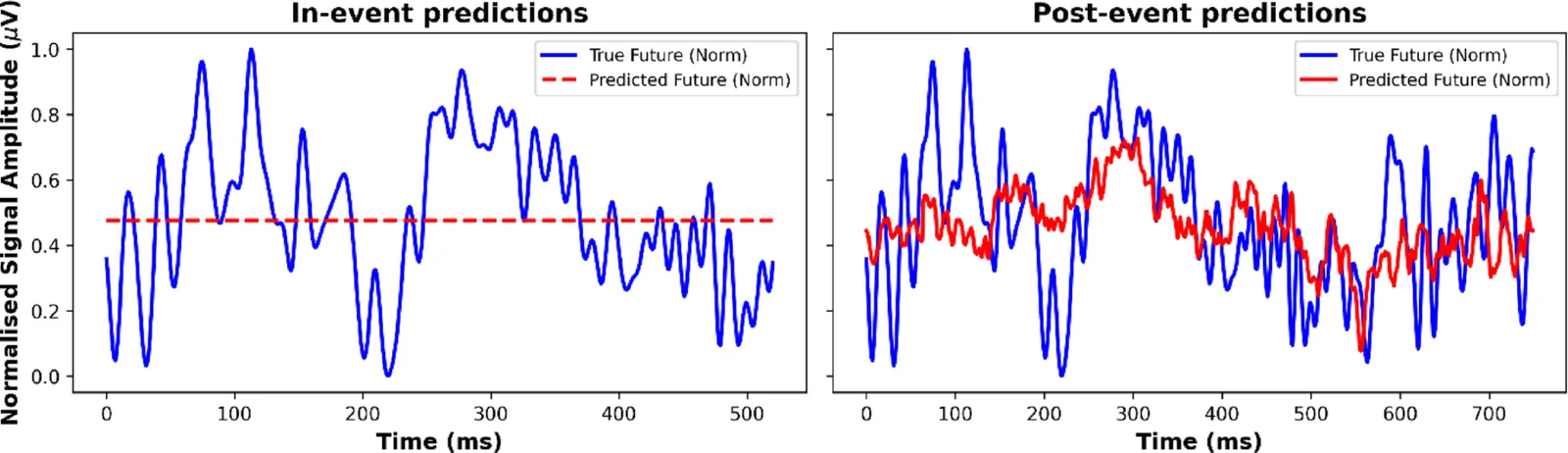
Quasi-null forecasts obtained during the event (left) and more meaningful forecasts obtained during the follow-up using a composite loss function (right)

Training WaveNet with the MSE loss yielded no meaningful forecasts, as shown in the left panel of Fig. 5. The follow-up training with a composite loss function gave more encouraging results, with forecasts capturing most of the slow-frequency and some of the high-frequency dynamics in the signal (as can be seen in the right panel).

### Discussion

Our results emphasize the complexity of learning causal temporal relationships in the non-band-filtered EEG signal. However, the net improvement observed during the post-event follow-up suggests that the problem can be solved in principle. Penalising large amplitude mismatches and temporal shifts was enough to capture many of the relevant signal dynamics, suggesting that incorporating domain knowledge in the training process could be beneficial. The Brainhack Rome event was fundamental to taking the project from the drawer to the testing bench. Future work will fine-tune the model’s architecture and loss function to achieve increasingly accurate forecasts.

### Data and code availability

The WAY-EEG-GAL dataset and the code developed in the project are publicly available (De Matola 2026; Luciw et al. 2014a).

## MULTIFUN: multicontrast multiscale analysis of functional brain networks

*Angelina Catrambone*,* Daniele Cirulli*,* Marida De Maria*,* Ottavia Falconi*,* Maria Guidi*,* Giulio Iannelli*,* Margherita Premi*,* Pablo Villegas*.

### Background

Functional magnetic resonance imaging (fMRI) based on the blood oxygenation level dependent (BOLD) is the most used imaging technique to study brain function. BOLD has a higher sensitivity compared to the other functional acquisitions but does not deliver quantitative information. Other contrasts, such as vascular space occupancy (VASO) and arterial spin labeling (ASL) give information on quantitative parameters and are directly connected to brain metabolism, thus offering the possibility to obtain complementary brain network information. Moreover, even within a single contrast, different frequency bands could carry information with different degrees of specificity to e.g. neuronal activity, vascular dynamics, and endogenous fluctuations in blood CO_2_.

Finally, the brain is inherently a multiscale system, with coordination spanning from circuit-like motifs to large-scale interactions between distributed areas. Therefore, relevant patterns may emerge at different resolutions and cannot be captured by a single clustering level (Betzel & Bassett, 2017). In this project, we aimed at identifying relevant characteristic frequencies of multicontrast functional timeseries and analysing their network behavior.

### Methods

Thirteen (4 male; age mean/SD: 60 ± 7 years) healthy participants were recruited at Fondazione Santa Lucia in Rome and were scanned on a Siemens MAGNETOM Prisma scanner (3T). Data acquisition was in accordance with the ethical standards of the Helsinki declaration and its later amendments. The study protocol was approved by the local ethics committee (approval 2022_010) and written informed consent was obtained from all participants prior to the study. The MRI protocol included three functional runs based on BOLD, VASO and ASL contrasts. The BOLD run was acquired in resting-state condition, while VASO and ASL during a breathing manipulation task (alternation of normocapnia and hypercapnia every two minutes). Repetition time (TR), number of measurement (N) and nominal resolution were:

- BOLD (resting-state): TR = 0.98 s, *N* = 488, (2.8 mm)³;
- VASO (task-based): TR = 2.7 s, *N* = 444, 2 × 2 × 3 mm³;
- ASL (task-based): TR = 5.5 s, *N* = 110, (4.4 mm)³.

All datasets were corrected for motion using a rigid-body transformation. The BOLD time course was preprocessed using fMRIPrep (Esteban et al. 2019). The VASO time course was preprocessed using LAYNII (Huber et al. 2021), which included a temporal upsampling of the VASO time course by a factor 2 in order to apply the dynamic division approach for the mitigation of BOLD contamination. The VASO-derived BOLD contrast was obtained by splitting the time course in an interleaved fashion. The ASL time course was preprocessed using ExploreASL (Mutsaerts et al. 2020) for cerebral blood flow (CBF) quantification. All the resulting time courses were spatially normalized to the MNI-152 space. Using the Harvard-Oxford cortical atlas (http://fsl.fmrib.ox.ac.uk/fsl/fslwiki/Atlases*)*, 48 mean time courses were obtained for each subject and contrast and used for subsequent analysis.

#### Frequency analysis

Correlation matrices were computed for each functional contrast after bandpass filtering. Time series were filtered using FFT to isolate three physiological frequency bands: slow-5 (0.01–0.027 Hz), slow-4 (0.027–0.073 Hz), and a higher frequency band, termed s* (0.073–0.18 Hz) derived from the analysis described below. For each contrast-frequency combination, correlation matrices were calculated and averaged across subjects to enable comparative analysis of functional connectivity patterns across different temporal scales and imaging contrasts.

To investigate the frequency structure of the signals, we applied empirical mode decomposition (EMD). EMD decomposes a signal into oscillatory patterns and a residual trend that are directly derived from the data. These oscillatory patterns, called intrinsic mode functions (IMFs), are defined by symmetry and zero-mean envelope criteria (Huang et al. 1998). For the current analysis, EMD was implemented using the Python package *emd* (Quinn et al. 2021). From each signal we extracted the characteristic frequency of the IMFs.

To evaluate the contribution of the relevant IMFs, we computed correlation matrices in two ways: (i) using band-pass filtered signals covering the slow-5 band up to the s* range, and (ii) using only the IMFs whose characteristic frequencies fell within s*. These results were then compared with the conventional correlation matrices derived from slow-4 and slow-5 bands.

**Fig. 6:**
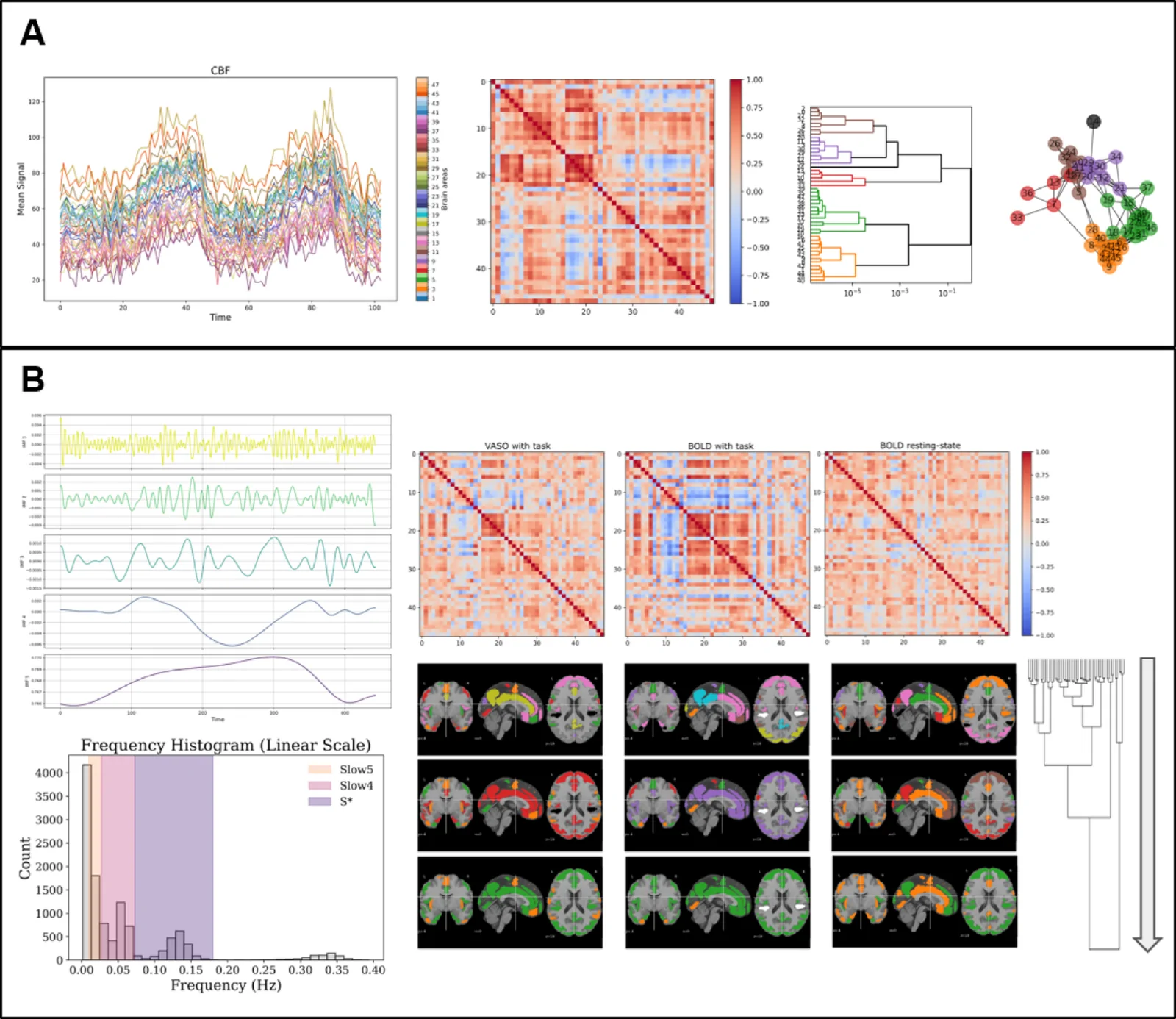
Panel **A** depicts the analysis workflow. The preprocessed time courses (left) were extracted from the ROIs defined by the Harvard-Oxford atlas; correlation matrices (center) were built for each functional contrast before and after filtering; networks (right) were then generated for each contrast and retained frequency. Panel **B** includes the resulting intrinsic mode functions (left image group) found using the EMD approach and the correlation matrices and maps of connected ROIs for the VASO and BOLD contrasts (right image group) for the frequency bands considered

The workflow is depicted in Fig. 6a.

#### Network analysis

To investigate the organization of brain activity across the different fMRI contrasts, we analyzed the functional connectivity (FC) networks derived from correlation matrices and their EMD components. These networks capture pairwise statistical dependencies between brain regions, providing a natural substrate for graph-theoretical analysis (Bassett and Sporns 2017). We employed a state-of-the-art approach for multiscale community detection, enabling us to track how modular structure evolves across scales and to assess which features are preserved or emphasized under different imaging contrasts. In particular, we rely on a Laplacian Renormalization Group (LRG) framework, a method grounded in the information flow of networks (Villegas et al. 2023). LRG defines coarse-graining schemes that preserve diffusion dynamics, producing intrinsic partitions of the network according to how information naturally spreads across its structure (Villegas et al. 2025) .

In this framework, the starting point is the graph Laplacian *L* = *D* – *A*, which encodes the diffusion operator of the network. From it, one defines a density matrix ρ(τ) as the exponential matrix of τ – *L*, where τ is the diffusion timescale. Entries of ρ(τ) matrix represent the probability of diffusing between two nodes in a time τ. The inverse of ρ(τ) can be interpreted as ultrametric distance *D* is introduced to quantify hierarchical similarity between nodes, providing the basis for community detection across scales. Thus the whole process simply requires diagonalizing the Laplacian matrix to compute the associated information communicability functions.

The computational counterparts and the dedicated pipeline for multicontrast applications have been publicly released (Iannelli et al. 2026).

### Results and discussion

Pooling data from all patients and contrasts, a histogram of the IMFs frequencies was constructed (Fig. 6B), which showed that, in addition to the established slow-5 (0.01–0.027 Hz) and slow-4 (0.027–0.073 Hz) bands, a substantial number of IMFs clustered at higher frequencies. We defined this additional range as s* (0.073–0.18 Hz). Applying the multiscale community detection framework to functional connectivity networks allows us to probe how modular organization unfolds across resolutions and how it differs between fMRI contrasts. Instead of committing to a single clustering level, the multiscale community detection framework allows us to explore the persistence, merging, or splitting of communities across scales (Fig. 6b).

Importantly, such multiscale analysis can be directly related to atlas-based parcellation: the degree of alignment or divergence between intrinsic, diffusion-driven partitions and standard anatomical boundaries offers a measure of how each contrast captures biologically meaningful subdivisions.

In conclusion, the EMD approach has proven capable of identifying characteristic features of signal modulation regardless of the contrast used, and the multiscale community detection framework has the potential for investigating how modular organization unfolds across resolutions and how it differs between fMRI contrasts.

## Conclusions

The Brainhack was successful in providing a venue for researchers from different backgrounds to propose and discuss interdisciplinary projects, leading to fruitful collaborations and promising results. Every project was characterized by the establishment of new connections among people who had not worked together before. The organizational efforts in terms of event promotion and program structuring facilitated an informal environment where learning from each other’s expertise was natural. Despite the overall success, there were still challenges, although most of them could be tackled. Reaching out researchers beyond one’s primary collaboration network can be tough, but we observed that a diverse organization team is a good starting point to form a heterogeneous audience. The interdisciplinary level of each project was good – at least one member in each project had different background and interests – but that could be improved, making the event reach a broader audience. Two key steps for such an improvement could be (1) more effective pre-event interactions, which require more planning in terms of platform choice and engagement strategies, and (2) a one-day hands-on workshop to provide a common starting point for all the participants. In the future, we aim to make this event a regular gathering to foster more cross-field endeavours, leveraging both cutting-edge neuroimaging techniques and rigorous complex systems tools to advance our understanding of brain structure and function.

## Data Availability

The datasets used for the sections “Adapting diffusion-weighted imaging algorithms to X-ray directional dark-field imaging”, “Developing a MATLAB toolbox for transfer entropy analysis: a data-driven approach to measuring effective connectivity with fMRI data” and “MULTIFUN: Multicontrast multiscale analysis of functional brain networks” are available upon request. The publicly available dataset “Structural and functional basis of neuronal plasticity” (https://zenodo.org/records/15149088) was used for the section “Connecting the dots between brain energy consumption and microstructure through latent space”. The publicly available WAY-EEG-GAL dataset (https://doi.org/10.1038/sdata.2014.47) was used for the section “I know what you will do: forecasting motor behaviour from EEG time series”. The publicly available Transdiagnostic Connectome Project dataset (https://doi.org/10.18112/openneuro.ds005237.v1.1.3) was used for the section “Hidden Markov Models and Task-based fMRI: Dynamic Brain Patterns of Emotional Processing across Psychiatric Disorders”.
